# Impact of implementation of a breech clinic in a tertiary hospital

**DOI:** 10.1186/s12884-020-03122-4

**Published:** 2020-07-29

**Authors:** S. Derisbourg, E. Costa, L. De Luca, S. Amirgholami, V. Bogne Kamdem, A. Vercoutere, W. H. Zhang, S. Alexander, P. M. Buekens, Y. Englert, A. Pintiaux, C. Daelemans

**Affiliations:** 1 Department of Obstetrics and Gynecology, Cliniques Universitaires de Bruxelles, Hôpital Erasme, Université Libre de Bruxelles (ULB), 808 route de Lennik, B-1070 Bruxelles, Belgium; 2 Research Laboratory for Human Reproduction, Faculty of Medicine, Université Libre de Bruxelles (ULB), 808 route de Lennik, CP 597, B-1070 Bruxelles, Belgium; 3 Perinatal Epidemiology and Reproductive Health Unit, Epidemiology, Biostatistics and Clinical Research Centre, Ecole de Santé Publique, Université Libre de Bruxelles (ULB), Bruxelles, Belgium; 4 Department of Epidemiology, School of Public Health and Tropical Medicine, Tulane University, New Orleans, Louisiana USA

**Keywords:** Breech delivery, Breech clinic, Care-pathway, Mode of delivery, Neonatal outcome

## Abstract

**Background:**

The incidence of breech presentation in single pregnancies at term is between three to 5 %. In order to support eligible women in their choice of mode of delivery, a dedicated breech clinic with a care pathway was developed in December 2015 in a tertiary referral centre in Brussels.

The primary objective of this study was to evaluate the vaginal birth rate before and after the introduction of a dedicated breech clinic. The secondary objective was to compare the early neonatal outcomes before and after the breech clinic was introduced.

**Methods:**

This was a single centre retrospective and prospective study. The inclusion criteria were term (from 37 weeks), singleton fetus and breech presentation at delivery. The exclusion criteria were suspected intrauterine growth restriction, severe fetal malformations and intrauterine fetal demise. We used a composite outcome as an indicator of neonatal morbidity and mortality.

**Results:**

After the introduction of the breech clinic, we observed a significant increase in planned vaginal delivery from 7.4% (12/162) to 53.0% (61/115) (OR: 13.5; 95% CI: 6.7–27.0). The effective vaginal breech delivery rate (planned and unexpected) significantly increased from 4.3% (7/162) pre-implementation of breech clinic to 43.5% (50/115) post-implementation (OR: 17.0; 95% CI: 7.3–39.6). Neonatal outcomes were not statistically different between the before and after periods.

**Conclusion:**

The introduction of a dedicated breech clinic has led to an increase in vaginal deliveries for breech babies without adversely affecting neonatal outcomes.

## Background

Breech presentation occurs in three to 5 % of singleton pregnancies after 37 weeks of gestational age (GA) [1]. The publication of the Term Breech Trial (TBT) in 2000 [2] had a significant impact on obstetric practice with many countries and international organisations recommending against vaginal birth. However, more recently, and mostly due to concerns surrounding the global rise in caesarean section rates and on-going criticisms about the methodology and the interpretation of the TBT, this option has been re-evaluated. The International Federation of Gynecology and Obstetrics (FIGO) [3], Le Collège National des Gynécologues et Obstétriciens Français (CNGOF) [4], the Royal College of Obstetricians and Gynaecologists (RCOG) [1] and the Society of Obstetricians and Gynaecologists of Canada (SOGC) [5] now support the option of vaginal breech birth [4, 6, 7].

The Cochrane Review of 2015 revealed a lower neonatal morbidity and mortality in the planned C-section group compared with the vaginal delivery group with an Odds Ratio (OR) of 0.3 and a 95% confidence interval (95% CI) of 0.2–0.6. However the TBT contributed to the majority of cases to the meta-analysis [2]. Multiple criticisms of this trial have already been published with many suggesting that selection was biased and expertise in breech delivery was suboptimal in some participating centres [8, 9]. Perhaps of more significance, was the failure to demonstrate any statistically significant difference in childhood outcomes between the two modes of birth at 2 years of age [10].

The PREMODA [7] study conducted in Belgium and France used similar outcome criteria as the TBT [2] but the methodology was prospective observational rather than a randomised trial. There was no specific management protocol, but the items used as a basis for deciding the mode of birth were: ultrasound evaluation of fetal size and cephalic flexion, maternal pelvimetry, woman’s desire to attempt a vaginal birth. The elements for managing and monitoring the labour were: the obstetrician’s self-assessed expertise in vaginal breech and continuous electronic fetal monitoring. The outcomes of 2526 planned vaginal breeches were compared to the outcomes of 5579 planned C-sections in 174 units. Neonatal short-term morbidity (five-minute Apgar score under four, injuries and intubation) was more frequent in the planned vaginal group compared to the planned C-section group (OR 8.92 (95% CI 1.0–79.8), 3.90 (2.4–6.34) and 1.82 (1.08–3.06) respectively). No other difference was found [7]. However since the publication of the TBT, the C-section rate for breech presentation has drastically increased in Europe [11–14] and expertise in vaginal breech birth has withered away.

Arguments against the conclusions of the TBT [2], the positive results of the PREMODA study [7], and globally rising C-section rates, have led to a reconsideration of vaginal breech birth by various authorities [1, 4–6]. A secondary analysis of the TBT highlighted the beneficial effect of the presence of an experienced clinician at breech births [15]. The recently published guideline by the RCOG [5] on the management of breech presentation suggests that ‘clinicians should counsel women in an unbiased way that ensures a proper understanding of the absolute as well as the relative risks of their different options’ and that ‘the presence of a skilled birth attendant is essential for safe vaginal breech birth’ [1]. In Belgium, the French-speaking Belgian guidelines [16] never ceased to recommend “selected vaginal breech” approach. However, compared to other European countries with the same policy, the national rate of vaginal birth in 2015 according to Euro-peristat [17] was low: 10.3% in Belgium, compared to 25.2% in France, a country with very similar guidelines, or 34.5% in Norway [17].

A returned focus on vaginal breech deliveries needs to be well planned. In order to support eligible women in their choice of mode of delivery, and to standardise care and counselling [18], a ‘breech clinic’ with a dedicated care pathway and a vaginal breech protocol were developed in our unit starting from December 2015. In order, to effectively support women and clinicians, a 24-h on-call specialist team was also established as suggested by Walker et al. [18, 19].

The primary objective of this hybrid retrospective and prospective study was to compare the planned and observed vaginal delivery rates before and after the implementation of the breech clinic. The secondary objective was to compare the early neonatal outcomes.

## Breech clinic

### Setting up the breech clinic

The breech clinic was developed in Cliniques Universitaires de Bruxelles ‘Hôpital Erasme’ a tertiary referral centre and the academic hospital of the ‘Université Libre de Bruxelles’ (ULB) in Brussels, Belgium. The breech clinic was not developed following formal program theory techniques ante hoc. The model aimed to remediate poor adherence to Belgian breech guidelines and included: new skills development, a dedicated team and clinic, and a rota of on-call breech specialists. This type of intervention has already been reported for external cephalic version (ECV) in breech [20], but also for vaginal birth after C-section, including a trial [21], or management of early pregnancy bleeding [22]. Belgian breech guidelines [16], as in some Nordic countries, consider the appropriate pathway includes timely recognition and routine consideration of ECV. And in case of unsuccessful ECV a discussion with the parents should lead to a partnership decision of the most appropriate mode of delivery. Our philosophy is to promote physiological labour and delivery with as few medical interventions as is safely possible. The maternity has around 2000 deliveries per year and since 2014 has also developed the first alongside midwifery unit in Belgium ‘the Cocoon’ [23].

Prior to the introduction of the breech clinic, no structured pathway of care for women with a breech presentation existed. In December 2015, such a care pathway was developed with dedicated appointments and an information leaflet for women (Supplementary material 1) explaining the ECV, the different modes of delivery and our antenatal education program. Since May 2016, each component of the service has been fully functional.

### Professionals

Six obstetricians and two midwives are dedicated to the breech clinic. Four of the obstetricians are experienced but two of the younger obstetricians still need direct supervision [19]. Two of the six are responsible for in-depth counselling, perform the ECV and select eligible women. One is regularly involved in the teaching of midwives, obstetric trainees and other colleagues [19]. The two midwives are responsible for the more physical aspects of a planned vaginal birth: training in pushing, maternal positions and pain management. For the delivery, one or two of the six “breech” obstetricians are always present and all support each other [24]. All members must have a training update in breech delivery at least once every year (local or international training, simulation, local or international congress and local review of cases with birth videos and/or medical records).

### Care pathway

**Fig. 1:**
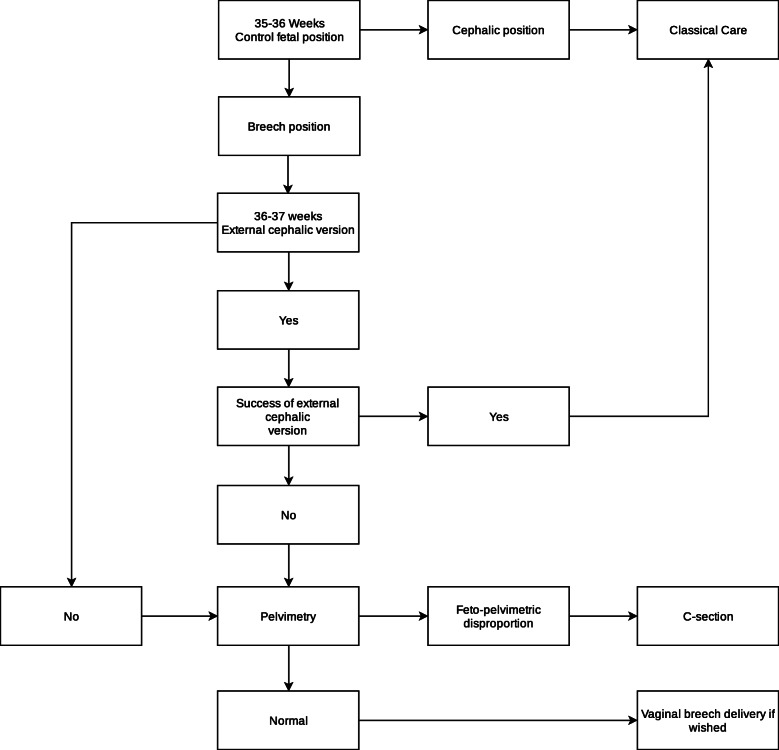
Care pathway of the breech clinic

If the fetus is in breech position at the routine ultrasound around 32 weeks of GA, the woman is referred to the breech clinic. An appointment is scheduled at around 35–36 weeks of GA to explain the ECV procedure, including the risks and benefits. If a woman opts for an ECV, this takes place at between 36 and 37 weeks of GA *(*Fig. 1*)*. One to three ECV are scheduled per week. In order to concentrate practice two of the obstetricians of the breech clinic perform it [25]. If the ECV is unsuccessful, in order to comply with the French-speaking Belgian college guidelines, a pelvimetry by computerized tomography (CT) scan is performed for all women [16] even though there is minimal evidence to support its use. As MRI-pelvimetry is less accessible in our service, most pelvimetries were performed using a CT-scan. Following the pelvimetry, an appointment is scheduled with one of the obstetricians from the breech clinic team to discuss the options for delivery, their relative risks and benefits and the management of labour, in case of a vaginal delivery attempt. Finally, if the woman opts for a vaginal delivery, we organise several sessions with a midwife in order to adequately prepare the woman for the labour and the delivery. All the women are offered the Erasme breech clinic leaflet (Supplementary material 1) containing information about ECV and breech delivery (vaginal delivery and C-section). The obstetricians work in accordance with the local guidelines and are comfortable and skilled at breech deliveries.

### Eligibility criteria for a breech delivery attempt

- Motivated expectant woman
- Estimated fetal weight above 2500 g
- GA more than 37 weeks
- CT-pelvimetry deemed adequate
- No cephalo-pelvimetric disproportion
- No other indication for C-section (two or more previous C-sections, placenta praevia, etc.)
- No hyperextension of the fetal head

### Management of labour and delivery

An ultrasound is performed at the time of the admission to the delivery suite to confirm the presentation and to exclude hyperextension of the fetal head. If indicated, induction of labour is performed ideally with favourable cervix (Bishop score > 6–7). Continuous electronic fetal monitoring is mandatory during the labour and if necessary, oxytocin and/or artificial rupture of membranes are/is used for augmentation. Epidural analgesia is available and used based on maternal request rather than systematically. During the birth, physiological techniques are preferred: no unnecessary manoeuvre is performed and the woman make their own decisions regarding birthing positions including, but not restricted to, upright or ‘on all fours’ positions [26]. Episiotomy is individualised and not performed systematically.

## Methods

### Study design

This was a single centre observational study, specifically designed to evaluate the impact of a dedicated breech clinic on vaginal breech births and on perinatal outcomes. Data were collected from the 1st of January 2013 to the 31st of November 2015 prior to the creation of the breech clinic and from the 1st of May 2016 to the 1st of October 2019 once the clinic was fully operational. From the 1st of March 2018, the patients were prospectively recruited and gave their informed consent to participate in the study. Prior to this date information was accessed using the medical records, without individual patient consent, with the approval of the local ethics research committee.

### Participants

**Fig. 2:**
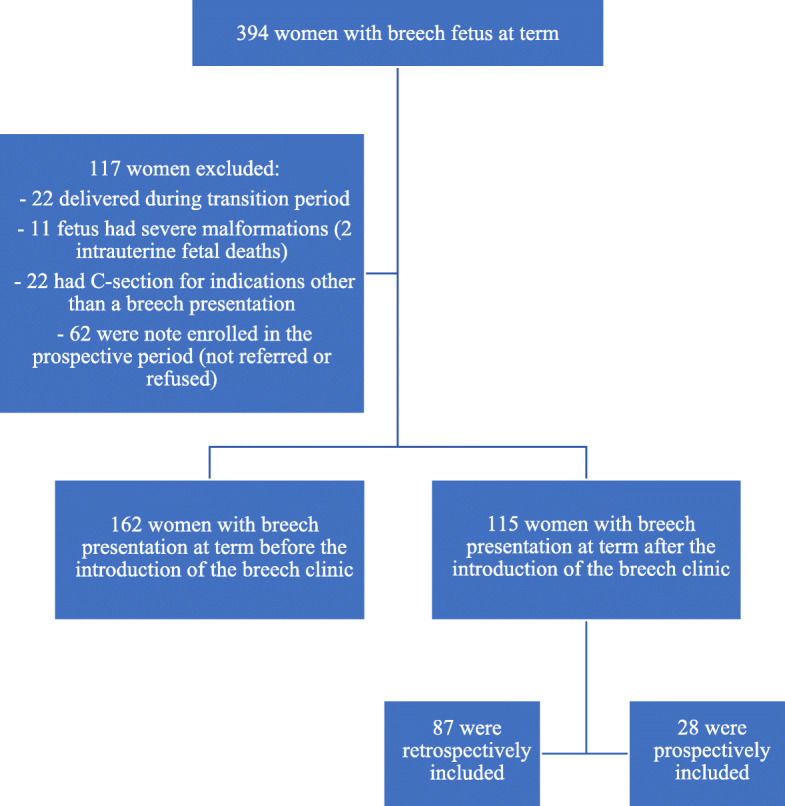
Flow chart of patients

The inclusion criteria were as follows: live term, singleton fetus in breech presentation at the time of delivery. Women who delivered during the transition period (5 months from the 1st of December 2015 until the 30th of April 2016) were excluded. Other exclusion criteria were: severe fetal malformations, intrauterine growth restriction (<10th centile) and intrauterine fetal demise or any obstetric contraindication to labour or vaginal delivery (e.g. placenta praevia or multiple previous C-sections) (Fig. 2).

### Data collection

The following variables were extracted from the hospital electronic database:

1. The baseline maternal characteristics: maternal age, body mass index (BMI), ethnicity (*Europe* for countries on the European continent, *sub-saharan Africa* for the African countries South of the Sahara, and *Mediterranean Basin* for the countries around the Mediterranean Sea except for France, Spain, Italy, and Greece, *Other* for the other countries or mixed race), assisted reproductive technology, smoking, parity, uterine scar, ECV attempts.
2. The mode of delivery: unexpected breech birth, planned and actual mode of delivery.
3. The neonatal outcomes: GA at birth, birth weight, 5-min Apgar score, arterial cord blood pH, arterial cord blood base deficit, neonatal intensive care unit (NICU) admission, parenteral and tubal feeding and duration, birth trauma, invasive ventilation support (intubation) or non-invasive ventilation support (continuous positive airway pressure for example) and duration, seizures, intracerebral ventricular haemorrhage, neonatal death.

### Outcomes

Our primary outcome was to compare the planned and actual mode of delivery before and after the introduction of the breech clinic. Our secondary outcome was to compare the short-term neonatal outcomes. A composite marker of perinatal outcome, which was similar to that proposed previously by the TBT [2] and the PREMODA study [7] was used (see Table 1 for the comparison of composite outcomes). This composite outcome is defined as one or more of the following: neonatal death, five-minute Apgar score under four, arterial cord blood base deficit above 15 mEq/L, neonatal trauma (brachial plexus injury or parietal skull fracture), NICU admission, NICU admission for over 4 days, parenteral or exclusive tubal feeding more than 4 days, seizures more than 24 h, intraventricular haemorrhage and intubation or non-invasive ventilation for more than 24 h.

**Table 1:** Comparison of the three neonatal composite outcomes

Term Breech Trial outcome	PREMODA outcome	Erasme breech clinic outcome
Fetal or neonatal mortality	Fetal or neonatal mortality	Fetal or neonatal mortality
Birth trauma including: - Subdural hematoma - Intracerebral or intraventricular haemorrhage - Spinal cord injury - Basal skull fracture - Peripheral-nerve injury present at discharge - Clinically significant genital injury	Birth trauma including: - Subdural hematoma - Intracerebral or intraventricular haemorrhage - Spinal cord injury - Basal skull fracture - Peripheral-nerve injury present at discharge - Clinically significant genital injury	Birth trauma including: - Subdural hematoma - Intracerebral or intraventricular haemorrhage - Spinal cord injury - Basal skull fracture - Peripheral-nerve injury present at discharge - Clinically significant genital injury
Seizures occurring at less than 24 h of age or requiring two or more drugs to control them	Seizures occurring at less than 24 h of age	Seizures occurring at less than 24 h of age or requiring two or more drugs to control them
Five-minute Apgar score of less than four	Five-minute Apgar score of less than four	Five-minute Apgar score of less than four
Cord-blood base deficit of at least 15	/	Cord-blood base deficit of at least 15
Hypotonia for at least 2 h	/	/
Stupor	/	/
Decreased response to pain, or coma	/	/
Intubation and ventilation for at least 24 h	Intubation and ventilation for at least 24 h	Intubation and ventilation for at least 24 h
Tube feeding for 4 days or more	Parenteral or tube feeding for at least 4 days	Parenteral or exclusive tube feeding for at least 4 days
Admission to the neonatal intensive care unit for longer than 4 days	Admission to the neonatal intensive care unit for longer than 4 days	Admission to the neonatal intensive care unit for longer than 4 days

We decided not to analyse the external cephalic version in this study.

### Analysis

Baseline maternal characteristics, rates of planned vaginal deliveries, rates of actual vaginal deliveries and neonatal outcomes before and after the implementation of the breech clinic were compared.

A chi-square test was used to compare categorical variables (ethnicity, smoking, parity, uterine scar, ECV attempts, the mode of delivery, neonatal death, 5 min Apgar score, arterial cord blood pH under seven, arterial cord blood base deficit more than 12 or 15 mEq/L, NICU admission, parenteral or tubal feeding and duration, intraventricular haemorrhage, non-invasive ventilation or intubation and the neonatal composite outcome). For continuous variables (maternal age, BMI, birth weight) a t-test was used after confirming that data was normally distributed. OR and the 95% CI were computed. A two-sided *p*-value of less than 0.05 was defined as statistically significant. Statistical analyses were performed using Stata version 12.

## Results

In total, there were 394 women with a fetus in breech presentation at the delivery from the 1st of January 2013 to the 1st of October 2019. A total of 117 women were excluded from further analysis: 22 delivered during the transition period, 11 had a baby with severe fetal malformations (two were intrauterine deaths), 22 had a C-section for indications other than a breech presentation and 62 were not enrolled for the prospective part of the study from the 1st of March 2018 (women were either not referred for enrolment or refused to participate). There were 162 women with a fetus in breech presentation at the delivery during the period prior the introduction of the breech clinic and 115 women since the dedicated breech clinic was in operation (Fig. 2).

There was no statistically significant difference between both groups concerning the baseline maternal characteristics except for the ECV rate (Table 2). The rate of ECV attempts increased from 63.6% before to 80.0% (OR 2.3; 95% CI 1.3–4.0) after the introduction of the breech clinic. There was no statistically significant difference between both groups concerning the labour characteristics of planned vaginal breech births (Table 3). A statistically significant increase in planned vaginal deliveries from 7.4 to 52.2% (OR 13.5; 95% CI 6.7–27.0) was observed after the introduction of the breech clinic. The total proportion of vaginal breech delivery (planned and unexpected) has statistically significantly increased from 4.3% (7/162) before implementation of the breech clinic to 43.5% (50/115) after the implementation (OR 17.0; 95% CI 7.3–39.6). Finally, a reduction in unexpected breech births from 9.3 to 3.5% was also observed (OR 0.3; 95% CI 0.1–0.9) (Table 4). Regarding the neonatal outcomes, there was no statistically significant difference before and after the start of the breech delivery pathway. However, we observed a slight trend towards a higher rate of arterial cord blood pHs < 7, of arterial cord blood base deficit > 12 mEq/L, and non-invasive ventilation > 24 h (Table 5). There was no case of invasive ventilatory support for more than 24 h or of non-invasive ventilation for more than 4 days. We observed less five-minute Apgar scores under four and under seven, less parenteral or tubal feeding longer than 4 days and less NICU admissions following the implementation of the breech clinic. Finally, there was no statistically significant difference between the two groups for the composite neonatal outcome. There were nine newborns with arterial cord blood pH < 7 and/or five-minute Apgar score under four whose details are listed in Table 6. Only one of these newborns spent more than 4 days in the NICU but required neither ventilation more than 4 days nor parenteral feeding. The two other babies who spent more than 4 days in the NICU had five-minute Apgar scores > 7 or normal arterial cord blood pHs at birth. One of the nine newborns, who was born by planned C-section, required parenteral feeding for more than 4 days. Lastly, the breech presentation was unexpected for three of the babies with an adverse neonatal outcome, all were born by emergency C-section.

**Table 2:** Baseline characteristics

	Before (***n*** = 162) n (%)	After (***n*** = 115) n (%)	***p***-value
**Maternal age in years**			
**< 21 years**	3 (1.9%)	0	0.34
**21–34 years**	115 (70.9%)	82 (71.3%)	
**≥ 35 years**	44 (27.2%)	33 (28.7%)	
**Body mass index**	(/162 observations)	(/113 observations)	
**< 18.5**	5 (3.1%)	5 (4.4%)	0.44
**18.5–24**	95 (58.6%)	69 (61.1%)	
**25–30**	34 (21%)	27 (23.9%)	
**> 30**	28 (17.3%)	12 (10.6%)	
**Ethnic origin**			0.77
**European**	64 (39.5%)	54 (47%)	
**Mediterranean Basin**	54 (33.3%)	32 (27.8%)	
**Sub-saharan Africa**	16 (9.9%)	3 (2.6%)	
**Other**	28 (17.3%)	26 (22.6%)	
**Assisted reproductive technology**	9 (5.6%)	8 (6.9%)	0.6
**Smoking**	21 (14.7%)	12 (11.9%)	0.53
	(/143 observations)	(/101 observations)	
**Primiparity**	99 (61.1%)	62 (53.9%)	0.23
**Uterine scar**	19 (11.7%)	8 (6.9%)	0.19
**External cephalic version**	103 (63.6%)	92 (80%)	0.003 OR: 2.3 (95% CI: 1.3–4.0)
**Gestational diabetes**	23 (14.2%)	18 (15.7%)	0.74
**Birth weight mean (± SD)**	3317.6 (**±** 432.1)	3253.8 (**±** 369.6)	0.20

*P*-value < 0.05 considered statistically significant. *OR* Odds Ratio; *CI* Confidence Interval; *SD* Standard Deviation; *BMI* Body Mass Index

**Table 3:** Labour characteristics of planned vaginal breech birth

	Before (*n* = 12) n (%)	After (*n* = 60) n (%)	*p*-value
Induction of labour	2 (16.7%)	6 (10%)	0.5
Epidural	10 (62.5%)	26 (40.6%)	0.74
Augmentation of labour	6 (50%)	23 (38.3%)	0.45
Expulsion efforts > 60 min	1 (12.5%)	11 (20.4%)	0.6
Episiotomy	4 (50%)	9 (30%)	0.29

*p*-value < 0.05 considered statistically significant

**Table 4:** Decision and mode of delivery before and after the introduction of the breech clinic

	Before (*n* = 162)	After (*n* = 115)	OR	95% CI
Unexpected breech delivery	15 (9.3%)	4 (3.5%)	0.3	0.1–0.9
Planned vaginal delivery	12 (7.4%)	60 (52.2%)	13.5	6.7–27.0
Effective vaginal delivery	7 (4.3%)	50 (43.5%)	17.0	7.3–39.6

*OR* Odds Ratio; *CI* Confidence Interval

**Table 5:** Neonatal morbidity and mortality

	Before (***n*** = 162)	After (***n*** = 115)	OR	95% CI
	n (%)	n (%)		
Five-minute Apgar score < 4*	2 (1.2%)	1 (0.9%)	0.7	0.1–7.8
**Five-minute Apgar score < 7**	5 (3.1%)	4 (3.5%)	1.1	0.3–4.3
**Arterial cord blood pH < 7**	1 (0.6%)	5 (4.4%)	7.3	0.8–63.5
**Arterial cord blood base deficit ≥ 12 mEq/L**	2 (1.2%)	6 (5.2%)	4.4	0.9–22.2
Arterial cord blood base deficit ≥ 15 mEq/L*	0	0	/	/
**NICU admission**	14 (8.6%)	8 (6.9%)	0.8	0.3–1.9
> 4 days*	2 (1.2%)	2 (1.7%)	1.41	0.2–10.2
**Parenteral or exclusive tubal feeding**	4 (2.5%)	5 (4.4%)	1.8	0.5–6.8
> 4 days*	1 (0.6%)	0	/	/
Birth trauma (severe hematoma)*	0	1 (0.9%)	/	/
**Intubation or ventilation**	9 (5.6%)	6 (5.2%)	0.9	0.3–2.7
> 24 h*	1 (0.6%)	2 (1.7%)	2.9	0.3–31.8
**> 4 days**	0	0	/	/
Seizures*	0	0	/	/
Neonatal death*	0	0	/	/
IVH grade I*	1 (0.6%)	0	/	/
**Composite neonatal outcome**	5 (3.1%)	4 (3.5%)	1.1	0.3–4.3

*OR* Odds ratio; *CI* Confidence interval; *NICU* Neonatal intensive care unit; *IVH* intraventricular haemorrhage. *Criteria included in the combined outcome “Composite neonatal outcome”

**Table 6:** Details of cases with adverse composite neonatal outcome

Cases	Breech clinic	Primiparous	Planned delivery	Actual delivery	C-section indication	2nd stage > 60 min	GA	Birthweight (in grams)	Five-minute Apgar score	Arterial cord blood pH	Arterial cord blood base deficit	NICU stay (in days)	Ventilation > 24 h and < 4 days	IVH grade 1	Parenteral feeding more than four days	Severe hematoma
**1**	No	No	Vaginal (Induction)	Vaginal	/	0	37.0	2520	**2**	7.15	9.8	0	No	No	No	No
**2**	Yes	Yes	Vaginal	Vaginal	/	1	40.2	3380	**3**	7.34	9.6	3	No	No	No	No
**3**	No	No	No plan	C-section	Breech presentation	NA	37.6	3130	**3**	7.25	8.7	0	No	No	No	No
**4**	Yes	Yes	Vaginal	Vaginal	/	0	41.2	3395	4	**6.99**	14.5	1	No	No	No	No
**5**	No	Yes	Vaginal	C-section (10 cms)	Acute fetal distress	0	39.5	2895	6	**6.97**	13.8	0	No	No	No	**Yes**
**6**	No	No	No plan	C-section	Breech presentation	NA	39.3	2940	9	7.31	2.6	**6**	No	**Yes**	No	No
**7**	No	Yes	C-section	C-section	Foeto-pelvic disproportion	NA	39.1	3070	10	Missing value	Missing value	**7**	No	No	**Yes**	No
**8**	Yes	Yes	No plan	C-section	Acute fetal distress	NA	39.1	3000	9	7.36	1.7	3	**Yes**	No	No	No
**9**	Yes	Yes	Vaginal	Vaginal	/	1	40.6	3260	5	7	14.3	**5**	**Yes**	No	No	No

Abbreviations: *NA* not applicable, *GA* gestational age; *NICU* neonatal intensive care unit; *IVH* intraventricular haemorrhage

## Discussion

This study has clearly demonstrated significant clinical differences for singleton breech presentations at term, before and after the introduction of a specialised breech clinic and care pathway. Prior to the development of the breech clinic the C-section rate for breech presentation at term was 95.7% probably as a consequence of the TBT [2], whose impact has been observed in several countries [14, 27–29]. The C-section rate for breech presentation at term after the introduction of the breech clinic was 56.5%. Using a specific management protocol for women with breech presentation, a statistically significant increase in proportion of planned and actual vaginal breech births and a statistically significant decrease in those unexpected were observed. Perhaps, more importantly, the increased rate of planned and successful vaginal breech births was not associated with a significant increase in adverse neonatal outcomes. However, we noticed there was a non-significant trend towards an increase in arterial cord blood pHs < 7 and arterial cord blood base deficit > 12 mEq/L. This trend may be explained by the observed significant increase in vaginal deliveries following the implementation of the breech clinic in a service where breech presentation was previously managed with C-section in 95% of cases. The overall rate of adverse neonatal outcomes was relatively high in both groups. This cannot be explained by differences in the maternal or labour characteristics between the two groups. However, breech presentation itself and not solely the mode of delivery appears to increase perinatal mortality and severe morbidity [30]. In the PREMODA study, breech presentation was associated with increased perinatal mortality and severe morbidity with odds ratios of 1.60 (95% CI 1.14–2.17) in the planned vaginal delivery group and of 1.45 (95% CI 1.16–1.81) in the planned C-section delivery group. Publications on the impact of equivalent breech services are scarce. Hejl et al. [31] also noticed an increased planned vaginal rate following the introduction of a specific protocol for breech presentations in their French level III maternity. Their rate of planned vaginal delivery increased from 32.7% in 2008 to 63.8% in 2014 (*p* < 0.05) with a reduction in their rate of emergency C-sections from 26.2 to 20% (p < 0.05). In another French level III University Hospital study, Michel et al. [32] reported similar results following the introduction of a special service protocol for breech presentations. They observed an increase in planned vaginal delivery from 30.0 to 44.5% (*p* < 0.01) and in successful vaginal deliveries for breech presentations from 24.0% in 2000–2004 to 38.5% in 2004–2008 (*p* < 0.001). Hejl et al. [31] also reported an increased rate of arterial cord blood pH < 7.1 from 1.8% (1/40) to 8.7% (8/92) (*p* = 0.04). NICU admission was required for 12.4% of babies after the introduction of the protocol, which is higher than our rate (6.9%). In contrast, Michel et al. [32] did not observe any statistically significant difference in neonatal outcomes before and after the application of their protocol. They reported an incidence of arterial cord blood pH < 7 of only 1.3% with their new protocol (compared to 4.4% in our study), 0.3% of 5-min Apgar score (compared to 3.5% in our study) and 0.1% of NICU admission. Their chosen composite neonatal outcome, similar to the one that used by the TBT [2] and the PREMODA group [7], at 0.7% was comparable between the two modes of birth (compared to 3.5% in our study) [32]. The rate of NICU admission decreased after the introduction of the breech clinic, nonetheless, this rate remains relatively high. However, it is important to note that the local threshold to admit newborns for surveillance in the NICU is low and admission per se is not necessarily an accurate indicator of either short or long-term outcomes. For example, the neonatal admission rate for term babies in Flanders (the northern part of Belgium) during 2018 was 11.1% [33]. A study in the United States showed that the neonatal admission rate at > 34 weeks is around 10% but varies greatly from 1.1 to 37% across NICUs and only 11% of those admissions were associated with high illness acuity criteria [34]. Low arterial cord blood pHs have been associated with more complications especially NICU admissions and seizures [35, 36]. Nonetheless some authors [37, 38] concluded that neonatal morbidity with an arterial cord blood pH < 7 can only be predicted when associated with low five-minute Apgar score and/or an important arterial cord blood base deficit. However, in the composite outcome used in our study which is inspired by Hannah et al. [2] and the PREMODA Study [7], the arterial cord blood pH was not used. In our series of newborns with adverse short-term perinatal outcomes only three of them had pH ≤7 (one pH = 7, two pH < 7) combined with a five-minute Apgar score under seven (none under four). Only one of these three babies, who was born after a planned vaginal delivery, had a long stay in neonatal intensive care (defined as more than 4 days and of 5 days in this case) and more than 24 h’ ventilation (but less than four days). None of them experienced seizures. Two of these had a prolonged second stage more than 60 min but only one stayed more than 4 days in the NICU with ventilation less than 4 days and no other complication. One of them, which was an unexpected breech delivery at term born by emergency C-section, suffered a grade I intraventricular haemorrhage. We cannot report any relationship between the short-term adverse neonatal outcomes. The implementation of a breech presentation pathway has led to a statistically significant decrease in unexpected breech births. This decrease is important as one third of the newborns with an adverse perinatal outcome were unexpected breech presentations. The proportion of non-significant adverse short-term outcomes that we have observed, will probably improve with increasing experience. We remain vigilant, even though there is no evidence in the literature that adverse short-term outcomes impact on longer-term outcomes. Ulander et al. report no difference in long-term outcomes between breech and cephalic babies born vaginally [39]. Two studies, including the TBT, compared the long-term outcomes of breech presentations born vaginally or by C-section and found no differences [10, 40].

In Europe, the Nordic countries also promote vaginal breech delivery however we found no reports of breech clinics in these regions. In 2010, Daviss et al. reporting on a national survey of Canadian maternities described a lack of enthusiasm for the creation of breech clinics. In this study, only one centre (out of 20 responders) considered that a breech clinic was feasible and desirable and two other hospitals considered forming a breech team [41]. Our breech clinic is the first one of its kind in Belgium, the results thus far are encouraging, and this gives us confidence and motivation to continue with this dedicated care pathway. Our intended purpose was to support women in their choice and in this regard, we have been successful. Kok et al. reported that 35% of women will choose a vaginal delivery for their breech babies and in terms of factors influencing their decisions, it was the two-years neonatal outcome that was the most important piece of information affecting mode of birth for the expectant mothers while the fathers were more strongly influenced by the maternal outcomes [42]. The concentration of vaginal breech deliveries in dedicated centres is effective not only to avoid unnecessary C-sections (because of practitioners lacking experience in breech delivery) but also to help improve the neonatal outcomes over time due to a growing expertise within the team.

The limitations of our study are the retrospective nature of the first part of the study and its lack of power, due to the sample size, to exclude differences in neonatal outcomes.

In this study, our objectives are not to demonstrate which factors are most important in avoiding a planned C-section or an emergency C-section for breech presentation. It is however plausible that our obstetricians and midwives, those directly working in the breech clinic and those not directly working in it, are now more confident in considering an attempt at vaginal birth, due to the existence of the clinic, the presence of an updated local protocol and the 24 h a day and 7 days a week on-call breech clinic dedicated obstetricians. As Walker et al. [19] suggest in their study, the expertise is developed by, among others factors, ‘confidence and competence among colleagues’ and the clinicians who have an interest in breech births should be supported within specialist teams. We also believe that the special preparatory sessions with doctors and midwives to discuss and plan their breech birth help to enhance women’s confidence in the breech delivery process.

## Conclusion

The implementation of a breech clinic in our hospital has led to a statistically significant increase in planned and observed vaginal deliveries for breech presentation at term without negatively impacting the rate of adverse neonatal outcomes.

## Supplementary information

**Additional file 1.** Leaflet of the breech clinic.

## Data Availability

The datasets used and/or analysed during the current study are available from the corresponding author on reasonable request.

## Abbreviations

TBT: Term Breech Trial
FIGO: International Federation of Gynecology and Obstetrics
CNGOF: Collège National des Gynécologues et Obstétriciens Français
RCOG: Royal College of Obstetricians and Gynaecologists
SOGC: Society of Obstetricians and Gynaecologists of Canada
OR: Odds Ratio
95% CI: 95% confidence interval
ECV: External cephalic version
ULB: Université Libre de Bruxelles
GA: Gestational age
CT: Computerized tomography
NICU: Neonatal intensive care unit
BMI: Body mass index
