# Supplementary material for: Impact of implementation of a breech clinic in a tertiary hospital
Source: BMC Pregnancy Childbirth. 2020 Jul 29;20:435. doi: 10.1186/s12884-020-03122-4 (PMC7391516; doi:10.1186/s12884-020-03122-4)
Supplement: Supplementary file 1 — Additional file 1. Leaflet of the breech clinic. [file 12884_2020_3122_MOESM1_ESM.pdf]

Hōpital Erasme

ULB

My baby is lying breech

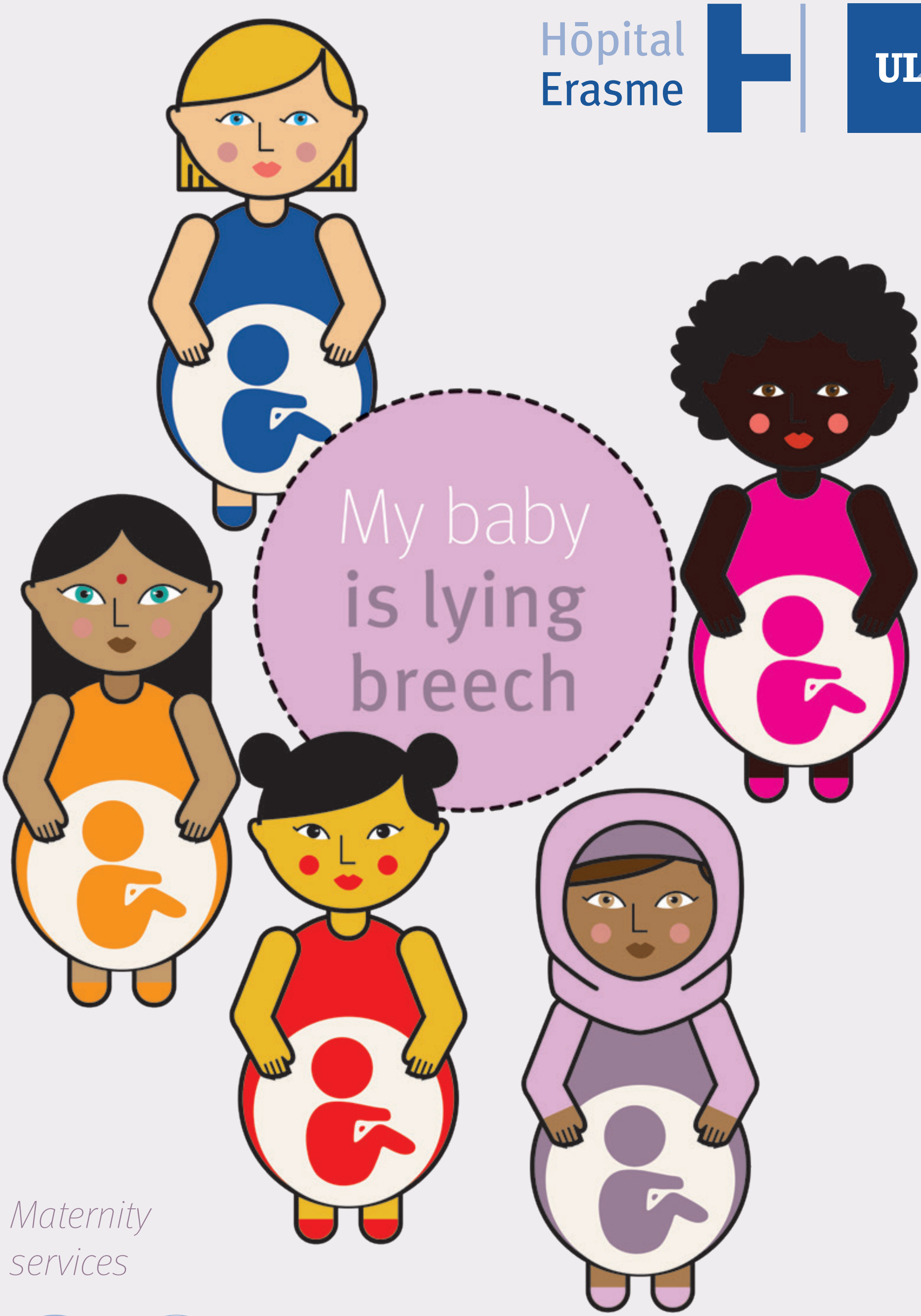

Maternity services

Contents

What does breech position mean?	3
The breech clinic	3
External cephalic version	4
Breech vaginal delivery	7
C-section	9
Practical information	10
My next appointments	11

What does breech position mean?

Breach position means that your baby is lying with its feet or its buttocks downwards in your pelvis. It is more common earlier in pregnancy. In the last trimester of pregnancy, most babies will turn to be lying head first.
After 36 weeks if your baby is still in breech position, the likelihood of spontaneous version to head down decreases. Around 3 to 5 % of babies will remain in the breech position around the due date.

The breech clinic

At 36 weeks if your baby is in breech position your carer will offer you an appointment at the Breech Clinic. The clinic runs

- In the delivery room **(route 316)**
- In fetal medicine **(route 178)**

During this appointment, the team will discuss your options with you:

- The possibility to try to turn it by an external cephalic version.
- An attempt at a vaginal breech birth or a planned C-section

If you are a suitable candidate for a vaginal birth and if you are willing to attempt it, you will have the opportunity to attend preparatory classes run by our specialised midwives, who will help you prepare for the birth of your baby at the breech clinic.

External cephalic version

This is an attempt to turn your baby to a head-down (head-first) position. The procedure is performed by one of the obstetricians involved in the breech clinic.

When? ECV is offered between 36 and 37 weeks. Occasionally it can be performed closer to the delivery if necessary.

Where? ECVs are performed in the delivery suite **(route 316)** or at the high-risk pregnancy unit **(route 323)** by one of the breech clinic obstetricians. Any person of your choice can support you during an ECV.

What are the chances of success? ECV is successful around 40% of the time. The success rate will be influenced by the amount of amniotic fluid around your baby, your baby's position in your pelvis, the position of its back and the position of the placenta.

Why is the ECV offered? If it is successful, you increase your chances of a vaginal delivery. However, it is important to note that a C-section remains necessary in some cases even if your baby is head first.

How is it performed?

Before the ECV

- A blood sample is taken before the procedure if it has not been performed earlier.
- We will check your baby's wellbeing with a fetal monitoring (recording of the heart of your baby) during 30 to 60 minutes.
- A drip is given for a medication that will help relax the uterus.
- You'll be asked to empty your bladder.

During the ECV

The procedure is performed with you lying flat on a bed. You may have a cushion to support your side or placed beneath your legs. It is important to be relaxed to facilitate the version.

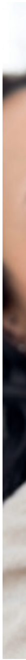

The doctor will apply pressure on your tummy with his/her hands to help your baby lift its buttocks out of your pelvis. This will encourage it to flip forwards or backwards. The procedure lasts a few minutes. A couple of attempts will be made (one to four) and we will check how your baby tolerates the procedure with ultrasound (monitoring its position and the activity of its heart).

The procedure can be interrupted at any time if you feel uncomfortable, you just need to tell the doctor.

At the end of the version, the position of your baby will be verified and another monitoring will be placed. If you are rhesus negative an injection of immunoglobulins (Rhogam®) is necessary and will be administered to you.

If the ECV is unsuccessful, you will be offered an imagery of your pelvis the same day.

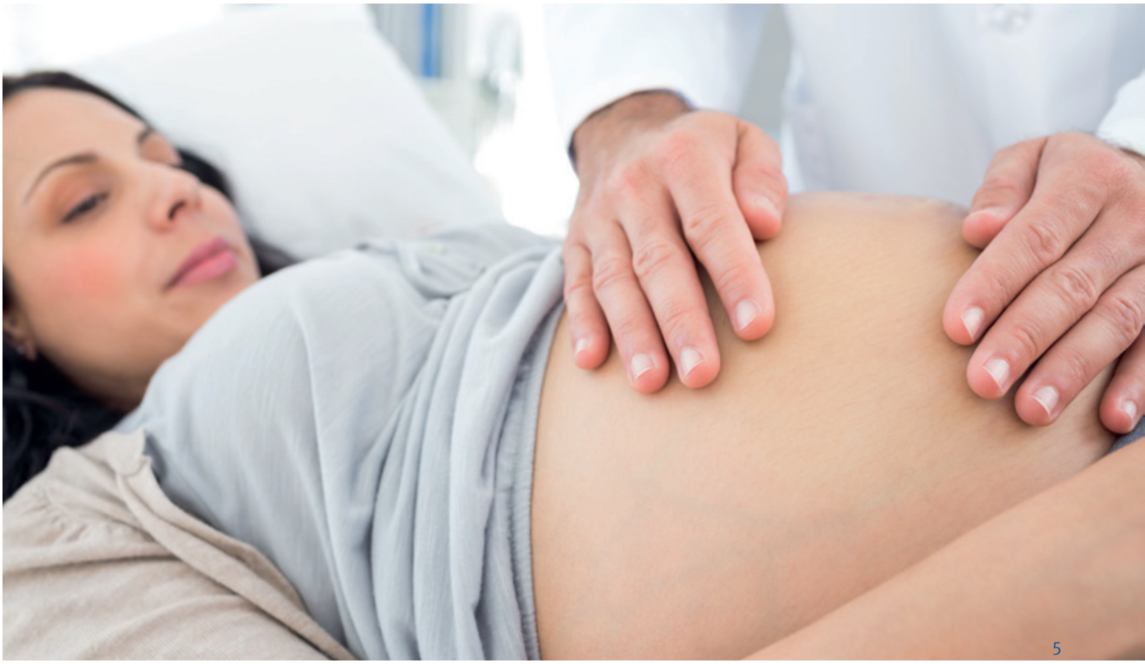

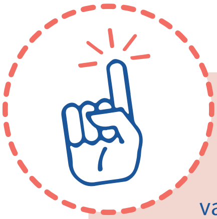

Is an ECV risky?

ECV is a routine medical procedure, without any complications in the vast majority of cases. However, as with all medical procedures, there is a degree of risk, and certain complications are possible [1]:

- Waters breaking (0,2%)
- Bleeding (0,3%)
- CTG anomaly (3,3%)
- In very rare cases (0,15%), a Caesarean section must be performed just after the ECV due to bleeding or a CTG anomaly.
- Pain for the mother (2,8%)
- Your baby may return to breech position even after a successful ECV (2,5%)

You could sometimes be advised to stay in hospital for the night for observation.

If once back at home, you are in pain or if you are bleeding or if you feel your baby less, **come back to see us on the labour ward (Route 316).**

It should be noted that these complications can also occur in patients who have not had an ECV, and that there are also risks if the procedure is not attempted, such as the risk of a Caesarean section being required, or the risks involved in a vaginal breech delivery.

Alternatives to ECV

Alternatives include osteopathy, acupuncture, and moxibustion (Chinese medicine). You can also try maternal positioning: this involves simple exercises used to encourage the baby to turn head down. These exercises will be explained to you at the unit.

(1) Statistics Hôpital Erasme

Breech vaginal delivery

70% of women will deliver vaginally if they opt for this route.

Observation

When you arrive on the labour ward, we will check how your baby is lying (position of the legs, flexion of the head) with ultrasound. A vaginal examination of your cervix will also be performed to determine how dilated your cervix is and how low the baby is lying.

During labour

Your baby's heart and your contractions will be monitored continuously during labour and delivery but this can be wireless so you can carry on moving around.

The epidural is not an obligation; you can choose if you want it or not. We can also suggest you take Kalinox® (mixture of gases) which gives you a short anesthesia. You have to breathe the gas in at the beginning of the contraction, then breathe normally during the rest of the contraction and it will help relax your body. You will need to stay in bed during this procedure but you can choose in which position. This product does not have any effect on your baby.

Whether or not you have an epidural, your midwife will encourage you to move and walk to help your baby descend into your pelvis. This decreases your risk of a C-section.

If labour progresses well, the cervix opens at a regular pace. If it is not the case, we might have to consider offering you a C-section.

For the delivery

You can opt for the birthing position of your choice. We tend to encourage the all fours position as it will give your baby the maximum space and allow the use of gravity to descend.

Once the baby's buttocks are low enough we will encourage you to push.

We might need to help you birth your baby. And it may be necessary to ask you to change position. The doctor might have to use specific maneuvers to help the baby to come out faster.

An episiotomy is not routinely performed.

Delayed clamping of the cord is offered if your baby is well.

What are the risks of a breech vaginal delivery?

For your baby:

- Immediately after birth, your baby may appear less alert, recovering from its birth.
- Its 1 minute Apgar score will typically be lower than a baby born head first.
- Your baby might require help with its breathing or neonatal care support.
- Its bottom can be bruised; its legs might remain extended for a couple of days.
- It may suffer from a dislocated hip. Its hips will be examined after birth by the paediatrician and an ultrasound of the hips will be offered usually 3 weeks after birth.

For you :

The risk of C-section during labour is higher (around 30%) than when the baby is head first.

C-section

If you opt for a C-section, this will be organised after 39 weeks.

Before the C-section

A blood test will be done if it hasn't been done before and you will meet the anaesthetist.

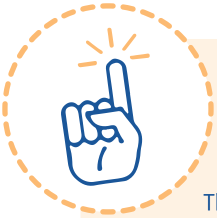

What are the risks of a breech C-section?

For your baby:

There is slightly less risk for the breech baby when born by C-section than when born vaginally. The difference is not statistically significant. Just like after a vaginal delivery:

- Its 1-minute Apgar score, used to assess a newborn's wellbeing, is usually lower than for a head-down baby.
- The baby's bottom can be bruised and the legs can remain extended for a couple of days.
- It may suffer from a dislocated hip. Its hips will be examined after birth by the paediatrician and an ultrasound of the hips will be offered usually 3 weeks after birth.

For you:

- In the short term, there is a higher risk of a blood clot developing in your legs, of bleeding and of infection. You will receive an injection to prevent a blood clot.
- The hospital stay is longer because you will need more time to recover.
- In the long term, there are risks for subsequent pregnancies, i.e. there is more risk of having another Caesarean section, as well as risks for your future baby and its placenta.

Practical information

This brochure covers all the most important information. We are of course fully at your disposal to answer any other questions you may have. For any questions or to change an appointment, please contact us on 02 555 33 25 or by email: siege.clini-obs@erasme.ulb.ac.be.

In an emergency, you can contact the delivery suite on 02 555 33 21.

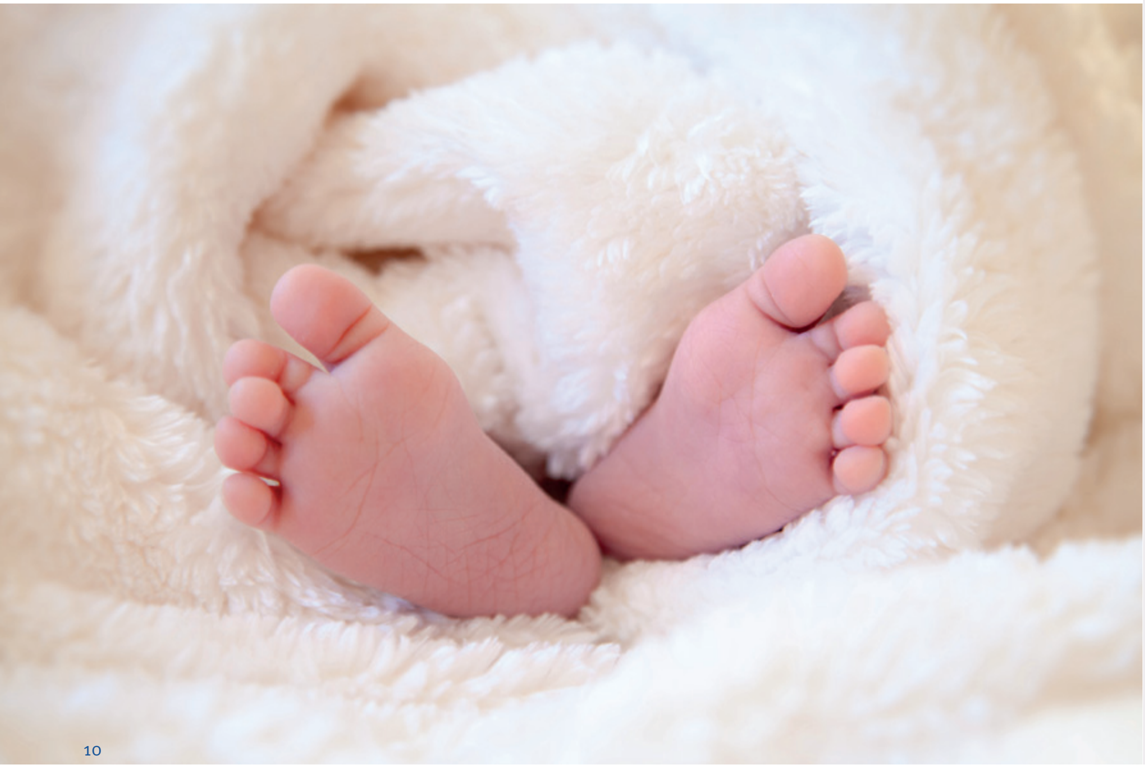

My next appointments

Breech clinic appointments with Dr ....

-
-
-

ECV date: ....

CT Scan of the pelvis: ....

Discussion about delivery method: ....

Birth preparation appointments with ....

-
-
-
-
-

My questions

....
....
....
....
....
....
....
....
....
....
....
....
....
....
....
....
....
....
....

« Why would one wish for a long life other than to help as many others as possible? » ERASMUS (1466-69 – 1536)

Hôpital Erasme
Route de Lennik 808 - B - 1070 Bruxelles
T 02 555 31 11 - **M** contact@erasme.ulb.ac.be

Maternity unit
Breech clinic
T 02 555 33 25 - **M** siege.clini-obs@erasme.ulb.ac.be.

www.erasme.ulb.ac.be

This brochure is also available in Dutch and French

Published by: Pr Johan Kips
Hôpital Erasme ULB - Route de Lennik 808 - B - 1070 Bruxelles
10/2018 • 28474/0
